# Earlier diagnosis of patients with brain tumour

**DOI:** 10.1136/pn-2025-004768

**Published:** 2025-09-18

**Authors:** Thomas C Booth, David Summers, Jagrit Shah, Helen Bulbeck, Robin Grant

**Affiliations:** 1 School of Biomedical Engineering and Imaging Sciences, King’s College London, London, UK; 2 King’s College Hospital NHS Foundation Trust, London, UK; 3 Department of Clinical Neurosciences, Royal Infirmary of Edinburgh, Edinburgh, UK; 4 Radiology Department, Nottingham University Hospitals NHS Trust, Nottingham, UK; 5 brainstrust, Cowes, UK

**Keywords:** TUMOURS, MRI

 This editorial outlines the recent expert recommendations for general practitioners on the early identification and referral of patients with possible brain tumours. Adults with progressive, subacute loss of central neurological function clearly need direct access to neuroimaging to assess for brain or central nervous system cancer. In the UK, since 2015 this has been recommended to be performed within 2 weeks of referral.^1^ However, we aim to raise awareness of the key diagnostic challenges in primary care because previous guidelines have not prioritised the importance of dual symptoms—such as headache and cognitive or personality change—which neurologists recognise as important when weighing up the likelihood of an intracranial cause.

It was shown over a decade ago that excessive waiting times and selection of cases for brain imaging from primary care contribute to the diagnostic delays of primary brain tumours, and one-third deteriorate clinically while awaiting brain scan appointments.^2^ Case selection of patients for urgent scanning is challenging because the presenting clinical features of brain tumours are broad and non-specific. Recently, neurology, neuroradiology, primary care, neurosurgery, ophthalmology and optometry experts developed recommendations for timely investigation.^3^ The recommendations highlight that recognising two symptoms occurring together as possible signs of a brain tumour allows patients to be referred for imaging sooner, thereby reducing the median waiting time from the first general practitioner (GP) visit to tumour diagnosis.^4 5^ Indeed, studies have shown that persons presenting with either headache, cognitive or non-specific neurological symptoms alone are slow to be diagnosed, whereas presenting with a headache together with additional symptoms, such as cognitive or personality change (i.e., headache ‘plus’), increases the likelihood of tumour on a scan. Importantly, assessing the value of performing a brief neurocognitive test (semantic verbal fluency test (SVFT): ‘how many animals can you think of in a minute?’) at the time of referral for scanning, identifies objective cognitive issues in those with headache suspicious of raised intracranial pressure and subtle cognitive symptoms that can be important when considering which patients with headache require urgent scanning.^6 7^

New headache alone is rarely due to tumour (0.1%), yet headache is the most common first presenting symptom (23%), and 46% of cases diagnosed in hospital have headache.^8^ A practical difficulty is that headache characteristics alone do not adequately differentiate those with a tumour from those without.^9^ Detailed symptom studies in patients with brain tumours note that subtle cognitive problems commonly precede headache but are not mentioned as they are attributed to the headache or to worry.^10^ Simple neurocognitive testing aids in the identification of cases without cognitive symptoms but with brain pathology.^11^ A position statement from the UK’s National Cancer Research Institute Brain Group (box 1) suggests that, in persons with headache suspicious of raised intracranial pressure, cognitive symptoms should be actively sought by directly asking the person or family member, and SVFT should be checked even in the absence of cognitive symptoms.^3 7^ An SVFT score of <17 animals in 1 min in someone with headache suspicious of cancer and subtle cognitive features increases the likelihood of finding tumour from 2.8% to >5% of cases. An SVFT score of <17 animals merits prioritising the urgency of neuroimaging for those with suspicious headache. This should use the modality with the shortest wait: either direct-access CT or MRI referral. A caveat is that, although CT is widely available and fast and can help with earlier diagnosis of a mass or hydrocephalus in such cases of headache ‘plus’, a normal CT scan cannot be completely reassuring in excluding a tumour cause, such as malignant meningitis or tumour-related venous sinus thrombosis. Here, further investigation should be considered if there is still suspicion of a cancer cause.

Box 1 **Referral to secondary care: a summary of the National Cancer Research Institute Brain Group position statement^3^**

**Emergency (same day) referral**

- Any person with ≥2 of these clinical features:
- Headache suspicious of cancer, new seizure, papilloedema and new focal neurological deficit.

Urgent referral suspicion of cancer *

- Any person with one of these clinical features.
  - Progressive neurological deficit—urgent standard of care—clinical discussion and imaging referral (MRI).
  - New adult-onset seizure—urgent standard of care—clinical discussion and imaging referral (MRI).
  - Headache suspicious of cancer and ‘uncertain discs’—optometry discussion and referral for ‘fields and fundi’.

Urgent referral suspicion of cancer*—(CT sufficient—if faster than MRI):

- Any person with a headache suggesting cancer plus one or more of the following features:
  - Cognitive change—symptomatic or noted by others or semantic verbal fluency test (SVFT) <17.
  - Personality change or SVFT <17.

*In the UK, urgent is defined as within 2 weeks.^1^

New-onset seizures over 18 years of age occur as a first symptom in about 20% of tumours and generally present quickly. An MR brain scan will identify infiltrative lower grade tumours more often than a CT scan and is the imaging of choice. Seizure ‘plus’ other neurological symptoms, or headache, increases the likelihood of a cause requiring urgent attention and should be discussed with neurology or acute medical services for ‘advice and guidance’.

Focal neurological symptoms, or signs, such as hemiparesis, hemisensory loss, dysphasia or visual field defects, collectively account for 20%–30% of first symptoms. Patients usually attend promptly, leading to early discussion and referral to secondary care. Focal neurological symptoms ‘plus’ headache merit urgent attention and should be discussed with neurology or acute medical services.

Optic disc swelling (papilloedema) can be difficult for the non-specialist to recognise. Only 14% have papilloedema and it almost always occurs with headache and other symptoms, for example, cognitive, focal neurological or seizure. For cases where the presence of papilloedema is uncertain, urgent assessment by optometry can speed up referral for assessment.

Cognitive symptoms, including poor attention, concentration or memory issues, are often initially attributed to benign causes, for example, poor sleep, and patients present late and are referred for imaging late. When additional symptoms, such as headache or focal symptoms, develop, referral should be expedited. Objective screening using SVFT can help.^11^

Patients unexplained neurological symptoms who have a history of cancer should always be considered for neuroimaging or discussed with oncology or acute medical services.

In 2023, the UK loosened referral guidance for suspected brain tumour and MRI, stating that ‘(primary care) should consider urgent direct-access tests…even if they do not think an urgent suspected cancer referral is appropriate’. The implication is that a large increase in direct referrals for MR brain scans from primary care is widely envisaged (alongside a decrease below 3% positive predictive value, which is the UK threshold used to warrant urgent scanning^5^). Non-protocolised acceptance of common symptoms, for example, headache, may lengthen waiting lists and paradoxically delay diagnosis in those few with a tumour. While the context is most useful within the UK, the evidence behind patient selection is relevant worldwide as the WHO implores: timely diagnosis is important for patient outcomes.^12^

## Data Availability

No data are available.
